# 3D-CAM: a novel context-aware feature extraction framework for neurological disease classification

**DOI:** 10.3389/fnins.2024.1364338

**Published:** 2024-02-29

**Authors:** Yuhan Ying, Xin Huang, Guoli Song, Yiwen Zhao, XinGang Zhao, Lin Shi, Ziqi Gao, Andi Li, Tian Gao, Hua Lu, Guoguang Fan

**Affiliations:** ^1^State Key Laboratory of Robotics, Shenyang Institute of Automation, Chinese Academy of Sciences, Shenyang, China; ^2^Institutes for Robotics and Intelligent Manufacturing, Chinese Academy of Sciences, Shenyang, China; ^3^University of Chinese Academy of Sciences, Beiing, China; ^4^Department of Radiology, The First Hospital of China Medical University, Shenyang, China; ^5^Department of Neurosurgery, Beijing Tiantan Hospital, Capital Medical University, Beijing, China; ^6^Shenyang Ligong University, Shenyang, China; ^7^Department of Neurosurgery, Affiliated Hospital of Jiangnan University, Wuxi, China

**Keywords:** medical image analysis, computer-aided diagnosis, deep learning, Parkinson’s disease, multiple system atrophy, regional Homogeneity, general feature extraction network

## Abstract

In clinical practice and research, the classification and diagnosis of neurological diseases such as Parkinson’s Disease (PD) and Multiple System Atrophy (MSA) have long posed a significant challenge. Currently, deep learning, as a cutting-edge technology, has demonstrated immense potential in computer-aided diagnosis of PD and MSA. However, existing methods rely heavily on manually selecting key feature slices and segmenting regions of interest. This not only increases subjectivity and complexity in the classification process but also limits the model’s comprehensive analysis of global data features. To address this issue, this paper proposes a novel 3D context-aware modeling framework, named 3D-CAM. It considers 3D contextual information based on an attention mechanism. The framework, utilizing a 2D slicing-based strategy, innovatively integrates a Contextual Information Module and a Location Filtering Module. The Contextual Information Module can be applied to feature maps at any layer, effectively combining features from adjacent slices and utilizing an attention mechanism to focus on crucial features. The Location Filtering Module, on the other hand, is employed in the post-processing phase to filter significant slice segments of classification features. By employing this method in the fully automated classification of PD and MSA, an accuracy of 85.71%, a recall rate of 86.36%, and a precision of 90.48% were achieved. These results not only demonstrates potential for clinical applications, but also provides a novel perspective for medical image diagnosis, thereby offering robust support for accurate diagnosis of neurological diseases.

## 1 Introduction

In clinical practice, Parkinson’s Disease (PD) and Multiple System Atrophy (MSA) are two neurodegenerative diseases. Despite their obvious differences in prognosis, treatment, and pathologic features, they are extremely similar in early symptoms (Palma et al., 2018). This poses a great challenge for doctors in their diagnosis (Song et al., 2007; Antonini, 2010). Parkinson’s disease has a high degree of heterogeneity, with different clinical subtypes, which makes diagnosis difficult (Wullner et al., 2023). According to statistics, the misdiagnosis rate of early-stage Parkinson’s disease can be as high as 20–30% (Poewe and Wenning, 2002). Misdiagnosis can potentially lead to doctors providing patients with incorrect treatment plans, resulting in disease progression and even irreversible neurological damage. Therefore, it is evident that diagnostic methods that rely solely on the personal experience of physicians may not be sufficiently reliable. As a result, there is an urgent need for a scientifically validated auxiliary diagnostic approaches to assist doctors in making diagnoses.

In recent years, with the development of medical imaging technology, many studies have been conducted to differentiate PD and MSA using advanced medical imaging. Among them, machine learning-based methods for extracting medical image features have shown promising results. For example, Chen et al. (2017, 2023) explored the differences in brain functional connectivity patterns between patients with PD and MSA, provided a diagnostic tool for PD and MSA using machine learning methods. Pang et al. (2020) extracted radiomics features on Susceptibility-weighted-imaging using machine learning methods for differential diagnosis of PD and MSA. Kim et al. (2022) constructed a machine learning model to extract radiological features using medical images, successfully differentiating various types of Parkinsonian syndromes. Bu et al. (2023) utilized different kinds of medical images to build a radiological model based on machine learning to differentiate PD from MSA. Although the above methods have shown good results in the diagnosis of PD and MSA, they all rely on manually selecting key feature slices and segmenting regions of interest. In addition, the features extracted by machine learning methods are filtered from a fixed set, which also presents limitations.

Currently, deep learning, as a cutting-edge technology of machine learning, shows great potential in the field of computer-aided diagnosis (Greenspan et al., 2016) and has made remarkable achievements in many aspects such as medical image analysis, pathology diagnosis and clinical decision support (Litjens et al., 2017; Panayides et al., 2020; Rehman et al., 2021). Some scholars have started applying deep learning techniques to studies on PD or MSA (Zhao et al., 2019; Jyotiyana et al., 2022; Wu et al., 2022). Among them, for the specific task of PD and MSA classification, some scholars have achieved considerable results by applying deep learning methods based on medical images. For example, Huseyn (2020) utilized Magnetic Resonance Imaging (MRI) with an improved AlexNet network structure to diagnose Parkinson’s disease, multiple system atrophy, and healthy individuals. Rau et al. (2023) proposed a deep learning algorithm capable of precisely segmenting the nucleus and shell, applying it to the diagnosis of PD and MSA. Compared to the aforementioned machine learning algorithms, although the features extracted by these methods are no longer limited to a fixed set of features, they still need to rely on manually selecting key feature slices and segmenting regions of interest, which does not allow for fully automated classification and diagnosis of diseases.

Therefore, we urgently need to develop a fully automatic classification model that can achieve classification diagnosis of PD and MSA without the need for manually selecting key feature slices and segmenting regions of interest. This approach would allow the model to comprehensively utilize data from the entire brain, enabling a comprehensive analysis of lesion features across various brain regions, thereby providing more reliable support for accurate diagnosis. In this study, we propose a novel 3D context-aware modeling framework called 3D-CAM. It allows a suitable convolutional neural network to be freely selected and embedded according to the dataset features in order to construct a classification model. The framework employs a 2D slicing-based strategy to process 3D Regional homogeneity (ReHo) data from brain Blood Oxygenation Level Dependent (BOLD) sequences (Zang et al., 2004). It segments the data into multiple 2D slices and uses them to train the classification model. The framework integrates two innovative modules: the Contextual Information Module and the Location Filtering Module. The Contextual Information Module is a feature enhancement module that can be inserted into any feature layer. It not only introduces features of adjacent slices, but also utilizes an attention mechanism to analyze the feature similarity between adjacent slices, enhancing focus on crucial features. This step effectively complements the inadequacy of 2D classification models in handling spatial information and contextual relationships. The Location Filtering Module is a post-processing module that not only leverages the 2D slice information to enable the model to concentrate on the slice segments with key features, but also analyzes and integrates the 2D slice information into the final 3D classification results. This step contributes to enhancing the model’s classification performance and enables it to identify key features more accurately.

The main contributions of this paper are as follows:

- A feature extraction framework called 3D-CAM is proposed for neurological disease classification. The framework achieves automatic classification with significant results in the classification tasks of PD and MSA.
- We propose a Contextual Information Module that can fuse the features of adjacent slices in any feature layer, enabling the network to emphasize key features and capture the spatial correlation between slices.
- We propose a Location Filtering Module that accurately concentrates on slice segments with key features, effectively enhancing the model’s classification performance by analyzing and integrating 2D slice information into 3D classification results.

## 2 Materials and methods

### 2.1 Dataset and preprocessing

The dataset for this study was obtained from the Neurology Outpatient Department of the First Hospital of China Medical University, covering patient data from July 2020 to August 2023.

For data acquisition, a 3.0 T MRI scanner outfitted with a 32-channel head coil was employed to acquire high-resolution T1 weighted MRI sequence and BOLD sequences in accordance with standardized scanning protocols. Subsequently, these sequences underwent processing to derive ReHo data. All processing procedures were executed using the Data Processing & Analysis of Brain Imaging (DPABI, RRID:SCR_010501), encompassing artifact removal, motion correction, temporal adjustments, and spatial normalization, as documented in pertinent literature (ChaoGan and YuFeng, 2010). Rigorous data quality assurance measures were undertaken, including meticulous data cleansing with manual exclusion of motion exceeding 3 mm, to ensure the integrity of the dataset.

**Figure 1:**
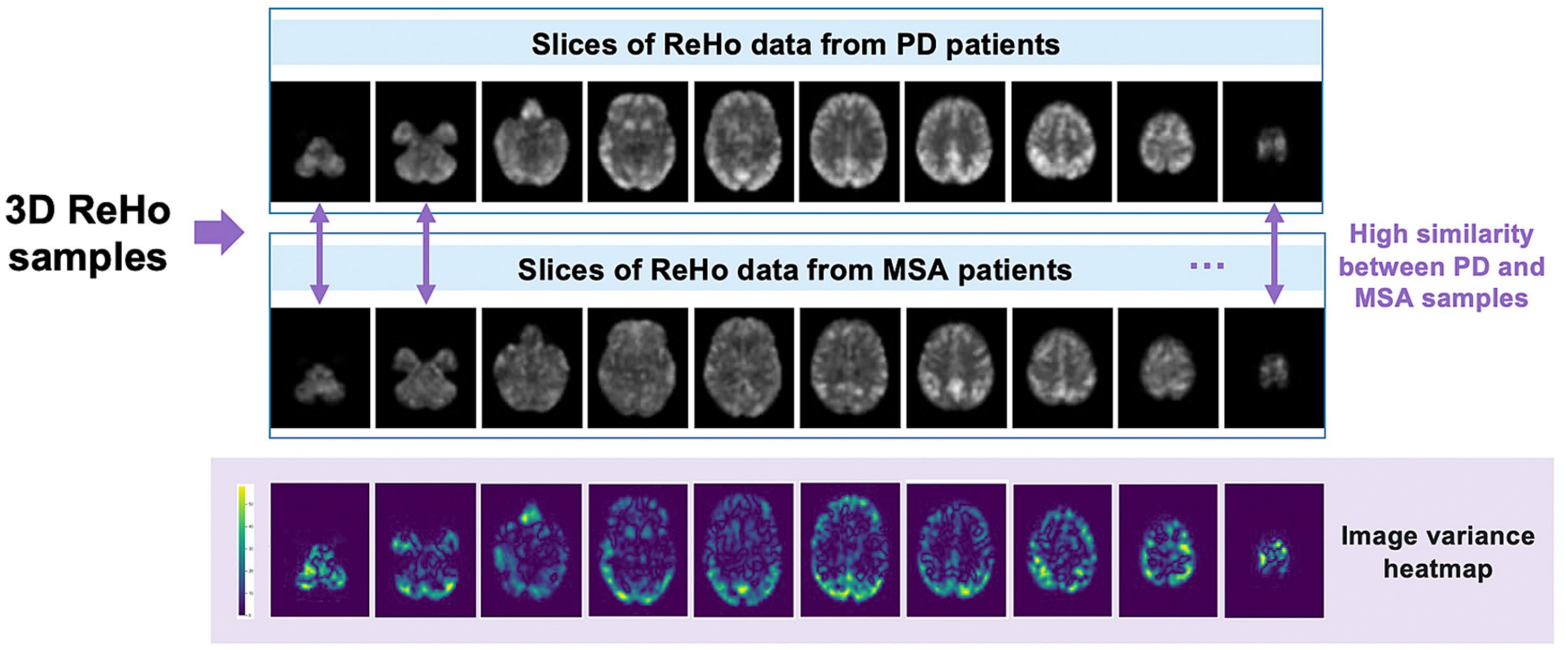
Example of ReHo data slices for PD patients and MSA patients.

Finally, we successfully obtained ReHo data from 189 patients, including 105 PD cases and 84 MSA cases. To ensure the effectiveness of model training, tuning, and evaluation, we divided these datasets into training set, validation set, and testing set according to the ratio of 7:1:2. Detailed sample information is shown in Figure 1.

### 2.2 Experimental setup and evaluation criteria

#### 2.2.1 Implementation

The classification models in this paper are programmed using Python and Pytorch. We performed all experiments on a personal workstation with an Nvidia GeForce RTX 3080 GPU. For the optimizer, we chose a learning rate of 0.0001, a weight decay of 0.0001, and used a stochastic gradient descent algorithm with a momentum of 0.9. The batch size for training was set to 32. In addition, we chose the cross-entropy loss function during the training process. This loss function is widely used in classification problems and can effectively measure the difference between the model output and the real labels, which helps to optimize the network parameters to improve the classification accuracy.

#### 2.2.2 Evaluation criteria

In evaluating the network’s classification performance, various evaluation metrics were introduced, including accuracy, precision, and recall. These indices are defined by Equations (1)–(3). Where TP, FN, FP and TN represent correctly classified positive samples, misclassified positive samples, misclassified negative samples and correctly classified negative samples, respectively. In this paper, positive samples are PD patient data and negative samples are MSA patient data.


(1) $$\mathrm{Accuracy}=\frac{TN+TP}{TN+TP+FN+FP}\times 100\%$$

(2) $$\mathrm{Recall}=\frac{TP}{TP+FN}\times 100\%$$

(3) $$\mathrm{Precision}=\frac{TP}{TP+FP}\times 100\%$$

In addition, we introduce the Receiver Operating Characteristic (ROC) curve and the Area Under the Curve (AUC). The ROC curve is a curve plotted with the True Positive Rate (TPR) as the vertical coordinate and the False Positive Rate (FPR) as the horizontal coordinate at different thresholds. The AUC value is the area under the ROC curve, which is used as a measure of the quality of the classifier’s prediction. The closer the AUC value is to 1, the better the performance of the classifier. In order to verify the robustness and generalization ability of the model, several experiments were conducted and the results were statistically analyzed and compared.

### 2.3 Methodology

#### 2.3.1 Overall framework

**Figure 2:**
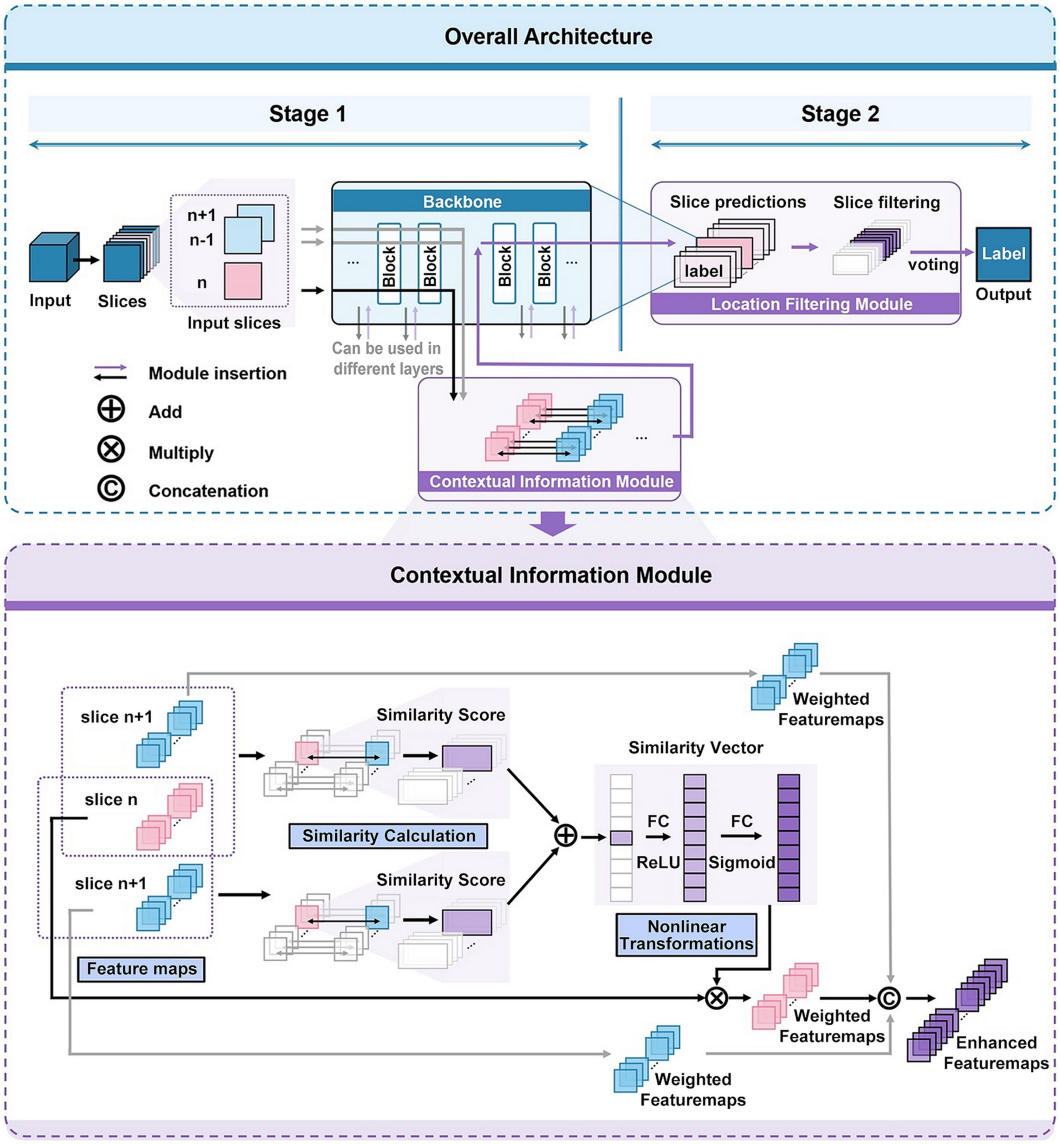
Network architecture of 3D-CAM. The upper part depicts the overall structure of 3D-CAM, including the specific configuration of the Location Filtering Module. The lower part depicts the detailed structure of the Contextual Information Module.

In this work, we propose a novel deep learning-based framework for 3D medical image classification, named 3D-CAM. The framework of 3D-CAM is illustrated in Figure 2. 3D-CAM employs a 2D slicing-based strategy to slice the 3D ReHo data into multiple 2D slices for putting into the network. Unlike traditional training methods, we incorporate several adjacent slices surrounding the current slice as inputs to the network. 3D-CAM can be divided into two main stages as follows.

In the first stage, we freely choose a suitable convolutional network, such as ResNet (He et al., 2016), and embed it into the 3D-CAM framework. As the convolutional network extracts features from the current slice layer by layer, it also simultaneously extracts features from adjacent slices. During the feature extraction process, we introduce a Contextual Information Module at an optimal location. This module can simultaneously receive feature maps of the current slice and its adjacent slices as inputs, thereby achieving the effect of introducing contextual information. In order to make the network focused on overall features and capture the spatial correlation between slices, we introduce attention mechanism in this module. By analyzing the similarity between the features of the current slice and its adjacent slices, we reassign weights to different features for feature enhancement. Afterwards, the enhanced features are further processed by convolutional networks to obtain the prediction results of 2D slices.

In the second stage, we introduce a Location Filtering Module to enhance the classification performance and integrate the 2D classification information into 3D classification results. This module is designed to filter the sliced segments with more significant classification features. In this step, we categorize all the 2D slice data used for training by location and compute the prediction accuracies of the slices with different locations in validation set. Based on this analysis, we filter out the consecutive slices with high prediction accuracy as the key prediction segments of the sample. Following this, we apply a voting mechanism to integrate the prediction results of each 2D slice within the segment to obtain 3D prediction results. This process aims to optimize and accurately extract segments with significant classification features serving as the basis for 3D classification.

#### 2.3.2 Contextual information module

When a 2D slicing-based strategy is used to process 3D data, the connection between different 2D slices is usually ignored, which leads to the loss of some spatial information. Therefore, to comprehensively capture data features and conduct a holistic analysis, we propose a Contextual Information Module, depicted in specific structure as illustrated in Figure 2.

When inserting a Contextual Information Module at the lth layer of a neural network with a depth of L to extract features, each input slice, following the initial l layers of the neural network, generates a collection of feature maps denoted as (4). $F_{i}^{\left(l\right)}$ denotes the set of feature maps obtained by extracting the ith input slice in the lth layer of the neural network. N denotes the number of feature maps in the lth layer, and $f_{i,j}^{\left(l\right)}$ denotes the jth feature map extracted from the ith input slice after passing through l layers of the neural network.


(4) $$F_{i}^{\left(l\right)}=\left\{f_{i,1}^{\left(l\right)},f_{i,2}^{\left(l\right)},\ldots ,f_{i,N}^{\left(l\right)}\right\}$$

When the network acquires $F_{i}^{\left(l\right)}$, its adjacent slices features $F_{i-1}^{\left(l\right)}$ and $F_{i+1}^{\left(l\right)}$ are also acquired at the same time and stored in the contextual feature module.

Next, to enhance the neural network’s attention to the overall features as well as to capture the spatial correlation between slices, we introduce attention mechanism. We compute the similarity between the feature map $f_{i,j}^{\left(l\right)}$ obtained from the current slice and the feature maps $f_{i-1,j}^{\left(l\right)}$ and $f_{i+1,j}^{\left(l\right)}$ of its adjacent slices. The Structural Similarity Index (SSIM) (Wang et al., 2004) is used here as a similarity measure. SSIM is an effective image similarity metric and its computation includes the consideration of statistics such as mean, variance and covariance. The SSIM formula (5) for calculating the similarity between two feature maps *A* and *B* is as follows:


(5) $$\mathrm{SSIM}\left(A,B\right)=\frac{\left(2\mu_{A}\mu_{B}+c_{1}\right)\left(2\sigma_{\mathrm{AB}}+c_{2}\right)}{\left(\mu_{A}^{2}+\mu_{B}^{2}+c_{1}\right)\left(\sigma_{A}^{2}+\sigma_{B}^{2}+c_{2}\right)}$$

In this formula, $\mu_{A}$ and $\mu_{B}$ denote the pixel mean values of feature maps A and B, $\mu_{A}^{2}$ and $\mu_{B}^{2}$denote their respective pixel variances, $\sigma_{\mathrm{AB}}$ is their pixel covariance, and $c_{1}$ and $c_{2}$ are constants introduced for stability.

Based on the aforementioned SSIM formula, we define the weight (6) as the average value of the similarity between the feature map $f_{i,j}^{\left(l\right)}$ and its adjacent slices of the feature maps $f_{i-1,j}^{\left(l\right)}$, $f_{i+1,j}^{\left(l\right)}$. The formula for this weight is as follows:


(6) $$\omega_{i,j}^{\left(l\right)}=\frac{\mathrm{SSIM}\left(f_{i-1,j}^{\left(l\right)},f_{i,j}^{\left(l\right)}\right)+\mathrm{SSIM}\left(f_{i,j}^{\left(l\right)},f_{i+1,j}^{\left(l\right)}\right)}{2}$$

In this formula, $\mathrm{SSIM}\left(f_{i-1,j}^{\left(l\right)},f_{i,j}^{\left(l\right)}\right)$ denotes the similarity between feature maps $f_{i,j}^{\left(l\right)}$ and $f_{i-1,j}^{\left(l\right)}$, while $\mathrm{SSIM}\left(f_{i,j}^{\left(l\right)},f_{i+1,j}^{\left(l\right)}\right)$ denotes the similarity between feature maps $f_{i,j}^{\left(l\right)}$ and $f_{i+1,j}^{\left(l\right)}$. The introduction of such weights aims to make the network paying more attention to the consistency of overall features while capturing spatial correlations between slices.

In order to better capture the correlation between feature maps, we specially design a neural network (7, 8) structure to dynamically adjust the original weight $\omega_{i,j}^{\left(l\right)}$. This structure comprises two linear layers and two activation functions. Here, $W_{1}$ and $b_{1}$ denote the weight matrix and bias terms of the first linear layer, and ReLU denotes the activation function of the first layer. $W_{2}$ and $b_{2}$ denote the weight matrix and bias terms of the second linear layer, and Sigmoid denotes the activation function of the second layer. The formulas are as follows:


(7) $$h_{i,j}^{\left(l\right)}=\mathrm{ReLU}\left(W_{1}.\omega_{i,j}^{\left(l\right)}+b_{1}\right)$$

(8) $${\omega_{i,j}^{\left(l\right)}}^{\prime }=\mathrm{Sigmoid}\left(W_{2}\cdot h_{i,j}^{\left(l\right)}+b_{2}\right)$$

Then, applying the adjusted weight ${\omega_{i,j}^{\left(l\right)}}^{\prime }$ to the original feature map, the weighted feature map (9) can be obtained.


(9) $${\hat{f}}_{i,j}^{\left(l\right)}={\omega_{i,j}^{\left(l\right)}}^{\prime }\cdot f_{i,j}^{\left(l\right)}$$

After that, each feature map in $F_{i}^{\left(l\right)}$ is weighted based on the previously mentioned steps to form a new set (10) of feature maps:


(10) $${\hat{F}}_{i}^{\left(l\right)}=\left\{{\hat{f}}_{i,1}^{\left(l\right)},{\hat{f}}_{i,2}^{\left(l\right)},\ldots ,{\hat{f}}_{i,N_{l}}^{\left(l\right)}\right\}$$

By introducing the attention mechanism, we adjust the weight of each feature map. The new set of feature maps ${\hat{F}}_{i}^{\left(l\right)}$ contains more spatially relevant and globally consistent features. It is conducive to better integrating spatial information into the feature extraction process, improving the perceptual ability and performance of the network.

In order to better incorporate the spatial information into the feature extraction network, we embed the feature maps $f_{i-1,1}^{\left(l\right)}$ and $f_{i+1,j}^{\left(l\right)}$ of adjacent slices together into the network, so as to comprehensively considering the contribution of adjacent slices features to the current features. We use weights α, β to perform weighted summation on the feature map sets $F_{i-1}^{\left(l\right)}$, $F_{i}^{\left(l\right)}$ and $F_{i+1}^{\left(l\right)}$ to obtain the final feature (11) after enhancement, where α is a smaller weight than β to emphasize the importance of the current slice. The formula of $W_{i}^{\left(l\right)}$ is:


(11) $$W_{i}^{\left(l\right)}=\alpha \cdot F_{i-1}^{\left(l\right)}+\beta \cdot F_{i}^{\left(l\right)}+\alpha \cdot F_{i+1}^{\left(l\right)}$$

This approach efficiently integrates the features of adjacent slices into the current feature maps, enhancing the capacity for global information representation of features.

After processing in the Contextual Information Module, we obtain a set of enhanced feature maps. These feature maps contain more spatial information and help the network to focus more on overall feature consistency while taking into account the importance of different features. Using similarity information to enhance the perception of spatial correlation can improve the feature representation capability of the network.

#### 2.3.3 Location filtering module

In order to improve the classification performance and integrate the 2D classification information into 3D classification results, this paper introduces a Location Filtering Module. In many 3D medical datasets, such as the dataset used in this paper, the slices at both ends usually contain less image information. Therefore, we want to filter the slice segments with more significant classification features located in the center location and vote them as key slice segments to obtain more accurate classification results.

First, we calculated the prediction accuracies of slices at different locations in the validation set. Let the number of 2D slices for each sample be N and the location number be i (from 1 to N). The accuracy of each location i is $Acc_{i}$ (12). Here, $\mathrm{Correc}t_{i}$ denotes the number of correctly predicted samples at location i, and $\mathrm{Tota}l_{i}$ denotes the total number of samples at location i.


(12) $$Acc_{i}=\frac{\mathrm{Correc}t_{i}}{\mathrm{Tota}l_{i}}\times 100\%$$

Next, we aim to find a contiguous segment among all possible slice segments where the average accuracy within that segment exceeds the threshold T. Additionally, this segment should be the longest among all possible segments, in order to retain as much information as possible while maintaining a high level of accuracy. This selected segment can be considered as the key slice segment and will be involved in subsequent voting and analyses. The formula to find this segment (13) is shown below:


(13) $$\left(S,L\right)={\mathrm{argmax}}_{j,k}\left(k\cdot 1\left(\overset{j+k-1}{\underset{l=j}{\sum }}Acc_{l}>k\cdot T\right)\right)$$

In this equation, j and k are parameters used to search for the longest segment. j represents the starting position, while k represents the length of the segment. $1\left(\cdot \right)$ is an indicator function that returns 1 if $\overset{j+k-1}{\underset{l=j}{\sum }}Acc_{l}>k\cdot T$ and 0 otherwise. Through this step, we finally identify the region of interest with a starting position S and length L with high prediction accuracy and use it as the key prediction segment.

Next, we adopt a voting mechanism to integrate the prediction results of each 2D slice located at the key slice segment aforementioned (14). The specific formula is as follows:


(14) $$P\left(c\right)=\frac{1}{N^{\prime }}\overset{N^{\prime }}{\underset{i=1}{\sum }}1\left(c_{i}=c\right)$$

In this equation, $P\left(c\right)$ denotes the probability that the final weighted voting prediction result is class c. $N^{\prime }$ denotes the total number of filtered slices in the key slice segment. $1\left(\cdot \right)$ is an indicator function, which indicates 1 when $c_{i}=c$, and 0 otherwise. $c_{i}$ denotes the prediction category of the ith slice. Finally, 3D prediction results can be obtained based on the prediction probability $P\left(c\right)$.

## 3 Results

In order to verify the reliability of the proposed method in this paper and to propose new methods for automatic diagnosis of PD and MSA, we conducted the following experiments.

### 3.1 Model selection and performance comparison

We applied our proposed innovative framework, 3D-CAM, to the classification tasks of PD and MSA. Specifically, we applied the two innovative modules, the Contextual Information Module and the Location Filtering Module, to specific layers of the classical model in order to enhance its performance. To ensure consistency, we inserted the Contextual Information Module in the layers corresponding to the 32 × 32 feature maps of each model. Additionally, we have investigated several families of classical convolutional neural network models, including EfficientNet (Tan and Le, 2020), DenseNet (Huang et al., 2018), ResNet (He et al., 2016), and Inception (Szegedy et al., 2015) as backbones. In order to identify the model with the best performance in our task, we compared multiple versions in each model family. Finally, we selected the best-performing model from each family for further analysis. The experimental results are detailed in Table 1.

**Table 1:** Comparative experimental results of different models.

Model	Accuracy	Recall	Precision
Inception	65.71%	68.18%	75.00%
Inception +3D-CAM	77.14%	77.27%	85.00%
DenseNet	68.57%	72.73%	76.19%
DenseNet +3D-CAM	80.00%	81.82%	85.71%
EfficientNet	71.72%	68.18%	83.33%
EfficientNet +3D-CAM	82.86%	86.36%	86.36%
ResNet	71.42%	72.73%	80.00%
**ResNet + 3D-CAM**	**85.71%**	**86.36%**	**90.48%**

The best-performing results are highlighted in bold.

In our experiments, we observe that different feature extraction networks can be embedded into 3D-CAM, while all of them show different degrees of improvement in classification accuracy. We also find that for our task, ResNet34-based 3D-CAM shows the best performance with 85.71% accuracy on the test set.

### 3.2 Ablation experiments

To verify the effect of our proposed two modules on the model performance, we conducted ablation experiments. On the currently best-performing model, we gradually removed these two modules and obtained two sets of ablation experimental results, as detailed in Table 2. Here, Module 1 represents the Contextual Information Module, and Module 2 represents the Location Filtering Module.

**Table 2:** Ablation experiments.

Model	Accuracy	Recall	Precision
ResNet	71.42%	72.73%	80.00%
ResNet + Module 1	74.29%	72.73%	84.21%
**ResNet + Module 1 + Module 2**	**85.71%**	**86.36%**	**90.48%**

The best-performing results are highlighted in bold.

The results of the ablation experiments showed that the removal of either module resulted in a significant decrease in model performance. This validates the importance of both modules to the model performance. These results strongly support the validity of our proposed modules and confirm their positive impact on the overall model performance.

### 3.3 Optimal insertion location analysis of the contextual information module

In order to determine the optimal insertion location of the Contextual Information Module, we conducted additional experiments. On the base of the currently best-performing model, we adjusted the insertion location of the Contextual Information Module and observed its effect on the model performance. We attempted to insert the modules at locations with different feature map sizes and recorded the optimal performance of the model performance in each case, as shown in Table 3.

**Table 3:** Experimental results inserted by the Module 1 at different locations on the optimal model.

Feature map size at the Module 1 insertion point	Accuracy	Recall	Precision
64 × 64	80.00%	81.82%	85.71%
**32 × 32**	**85.71%**	**86.36%**	**90.48%**
16 × 16	82.86%	81.82%	90.00%
8 × 8	74.29%	77.27%	80.95%

The best-performing results are highlighted in bold.

**Figure 3:**
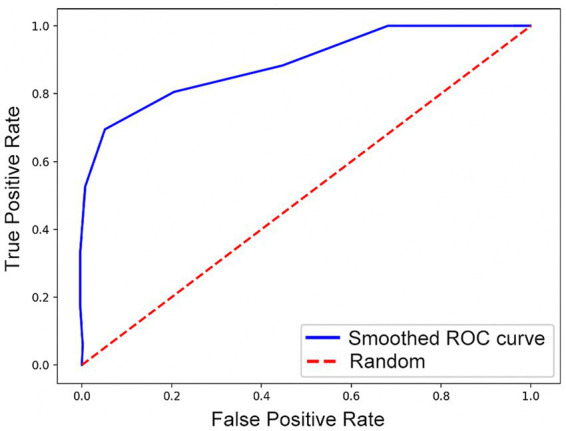
ROC curve.

The results above show that inserting the Contextual Information Module after a feature map of 32×32 size can bring the maximum performance improvement to the model with an accuracy of 85.71%. The ROC curve of the best-performing model is shown in Figure 3, with an AUC value of 0.85. This result also demonstrates the impact of different insertion locations of the Contextual Information Module on the model performance. These results strongly support the necessity of exploring the optimal insertion location for the Contextual Information Module and provide important ideas for improving the model performance.

In summary, through all the aforementioned experiments, we have successfully determined the optimal model for this task. This process not only validates the effectiveness of the proposed innovative modules, but also provides an effective method for the automatic classification of PD and MSA.

## 4 Discussion

Our experimental results demonstrate that the deep learning framework 3D-CAM can effectively classify PD and MSA based on medical images, achieving a classification accuracy of 85.71% and an AUC value of 0.85. This outcome shows the capability of our research method to learn disease-related image features from medical imaging data in an effective manner.

We speculate that the reason why the 3D-CAM framework performs well in experiments and outperforms other classical deep learning models is because it is specifically designed for PD and MSA classification tasks. It not only learns features from the current slice but also integrates key features from adjacent slices. At the same time, by utilizing attention mechanisms, it allows the network to focus more on overall features and the segment of slices that contain crucial features.

Recently, several studies have shown promising results in the diagnosis of PD and MSA using machine learning methods, such as Chen et al. (2017), Pang et al. (2020), Bu et al. (2023), and Chen et al. (2023). Among them, the dataset volume used by Pang et al. (2020) is comparable to our research, making it highly relevant. They obtained an AUC value of 0.862 in the classification task of PD and MSA on the test set, slightly higher than our result of 0.85. Although their results slightly outperforms the one we proposed, the approach by Pang et al. (2020) relies on manually selecting key feature slices and segmenting regions of interest, which greatly increases the subjectivity and complexity of the classification process. Moreover, the features extracted by machine learning methods are selected from a fixed set, which also presents limitations. The other studies based on machine learning methods mentioned above also suffer from these issues.

Some researchers have attempted to diagnose PD and MSA through deep learning methods, such as Huseyn (2020) and Rau et al. (2023). Among them, Huseyn (2020) proposed an innovative deep learning model, achieving an accuracy of about 88% in the classification task of PD and MSA, slightly higher than our proposed model’s accuracy of 85.71%. However, the aforementioned deep learning methods still rely on manually selecting key feature slices and segmenting regions of interest, which does not allow for fully automated classification and diagnosis of diseases. In addition, neurodegenerative diseases affect the entire brain of patients, and relying solely on local brain information within the regions of interest limits exploration of lesion features in other brain regions.

Compared to previous studies, 3D-CAM framework has achieved significant progress in the classification of PD and MSA. It no longer rely on manual selection of slices and regions of interest, successfully achieving fully automated classification. This method significantly reduces the investment of manpower and time. Additionally, by conducting direct analysis of global brain data instead of restricting to specific regions of interest, it enables the capture of more comprehensive feature information from the entire brain, leading to a significant enhancement in diagnostic accuracy and efficiency.

However, despite the promising results achieved by our approach, it is important to note some potential limitations. Firstly, although we have conducted our research using a large amount of data, the outcomes are still constrained by the current dataset. In the future, with the increase of data volume, we are expected to further optimize the model to obtain more reliable and comprehensive diagnostic results. Secondly, our study has focused solely on the classification tasks of PD and MSA, and applications to other neurological disorders have not been explored. Therefore, future research can further investigate the applicability of this framework in classifying other diseases.

In conclusion, our research has proposed an effective deep learning framework that offers a reliable solution for the classification of PD and MSA based on medical imaging, achieving satisfactory classification accuracy. This study offers strong support for early detection of neurodegenerative diseases and has broad prospect for clinical application. Additionally, our research provides new ideas and tools for the diagnosis and treatment of neurodegenerative diseases, and is expected to provide solid support for the future advancement of related fields.

## Data availability statement

The raw data supporting the conclusions of this article will be made available by the authors, without undue reservation.

## Ethics statement

The studies involving humans were approved by the Medical Research Ethics Committee of the First Affiliated Hospital of China Medical University. The studies were conducted in accordance with the local legislation and institutional requirements. The participants provided their written informed consent to participate in this study. Written informed consent was obtained from the individual(s) for the publication of any potentially identifiable images or data included in this article.

## Author contributions

YY: Writing – original draft, Writing – review & editing, Methodology, Project administration. XH: Writing – original draft, Writing – review & editing, Project administration. GS: Funding acquisition, Resources, Writing – review & editing. YZ: Funding acquisition, Resources, Writing – review & editing. XZ: Funding acquisition, Resources, Writing – review & editing. LS: Funding acquisition, Resources, Writing – review & editing. ZG: Funding acquisition, Resources, Writing – review & editing. AL: Funding acquisition, Resources, Writing – review & editing. TG: Funding acquisition, Resources, Writing – review & editing. HL: Funding acquisition, Resources, Writing – review & editing. GF: Supervision, Writing – review & editing.
